# Supplementary material for: Matching sensor ontologies through siamese neural networks without using reference alignment
Source: PeerJ Comput Sci. 2021 Jun 18;7:e602. doi: 10.7717/peerj-cs.602 (PMC8237319; doi:10.7717/peerj-cs.602)
Supplement: Supplemental Information 1 [file peerj-cs-07-602-s001.zip › 301/backup/refalign.html]

# (level 0) Alignment

## Source: http://oaei.ontologymatching.org/2011/benchmarks/101/onto.rdf

## Target: http://oaei.ontologymatching.org/2011/benchmarks/301/onto.rdf

## Correspondences

type = hasType
:   1.0

howPublished = howPublished
:   1.0

volume = hasVolume
:   1.0

annote = hasAnnotation
:   1.0

copyright = hasCopyright
:   1.0

Unpublished = Unpublished
:   1.0

address = hasAddress
:   1.0

chapter = hasChapter
:   1.0

Chapter < Inbook
:   1.0

editor = hasEditor
:   1.0

InBook = Inbook
:   1.0

series = hasSeries
:   1.0

date = hasYear
:   1.0

title = hasTitle
:   1.0

Booklet = Booklet
:   1.0

LectureNotes < Entry
:   1.0

url = hasURL
:   1.0

MastersThesis = Mastersthesis
:   1.0

mrNumber = hasMrnumber
:   1.0

pages = hasPages
:   1.0

TechReport = TechReport
:   1.0

edition = hasEdition
:   1.0

lccn = hasLCCN
:   1.0

affiliation = hasAffiliation
:   1.0

institution = hasInstitution
:   1.0

organization = hasOrganization
:   1.0

publisher = hasPublisher
:   1.0

school = hasSchool
:   1.0

Misc = Misc
:   1.0

collection < hasBooktitle
:   1.0

Collection < Book
:   1.0

isbn = hasISBN
:   1.0

abstract = hasAbstract
:   1.0

location = hasLocation
:   1.0

Article = Article
:   1.0

Informal < Entry
:   1.0

price = hasPrice
:   1.0

Book = Book
:   1.0

proceedings = hasBooktitle
:   1.0

PhdThesis = Phdthesis
:   1.0

Proceedings = Proceedings
:   1.0

number = hasNumber
:   1.0

Reference = Entry
:   1.0

InCollection = Incollection
:   1.0

author = hasAuthor
:   1.0

Report < Entry
:   1.0

note = hasNote
:   1.0

humanCreator = humanCreator
:   1.0

Part < Entry
:   1.0

Manual = Manual
:   1.0

key = hasKey
:   1.0

InProceedings = Inproceedings
:   1.0

journal = hasJournal
:   1.0

Monograph < Book
:   1.0

keywords = hasKeywords
:   1.0

issn = hasISSN
:   1.0

contents = hasContents
:   1.0

Deliverable < Entry
:   1.0

size = hasSize
:   1.0
